# GS143, an inhibitor of E3 ligase β-TrCP, reverses HIV-1 latency without activating T cells via unconventional activation of NFκB

**DOI:** 10.1371/journal.ppat.1013018

**Published:** 2025-04-01

**Authors:** Srijata Sarkar, Yoshifumi Kobayashi, Timothy Russnak, Jing Shen, Ronald G. Nahass, Joseph P. Dougherty, Céline Gélinas

**Affiliations:** 1 Center for Advanced Biotechnology and Medicine, Rutgers University, Piscataway, New Jersey, United States of America; 2 Department of Pharmacology, Rutgers Robert Wood Johnson Medical School, Piscataway, New Jersey, United States of America; 3 Graduate Program in Molecular Genetics and Microbiology, Rutgers School of Graduate Studies, Piscataway, New Jersey, United States of America; 4 IDCare, Hillsborough, New Jersey, United States of America; 5 Department of Medicine, Rutgers Robert Wood Johnson Medical School, New Brunswick, New Jersey, United States of America; 6 Department of Biochemistry and Molecular Biology, Robert Wood Johnson Medical School, Rutgers University, Piscataway, New Jersey, United States of America; Loyola University Chicago, UNITED STATES OF AMERICA

## Abstract

HIV-1 persists indefinitely in individuals living with HIV-1 even after effective treatment with antiretroviral therapy (ART). Upon cessation of the therapy, latently infected memory CD4+ T cells allow for a rapid rebound of the virus. The development of latency reversing agents (LRAs) to activate latent virus promoting immune recognition and clearance of the infected cells is pivotal for the elimination of the latent arm of the infection. Success of this strategy requires the development of potent highly specific LRAs with fewer off-target effects. LRA activity displayed by proteasome inhibitors although not highly specific opens the possibility of exploiting the high degree of specificity of the ubiquitin-proteasome system to develop targeted LRAs. Here we demonstrate that a small molecule GS143, which inhibits β-TrCP, the substrate recognition subunit of the SCF^*β*-TrCP^ E3 ubiquitin protein ligases, exhibits potent LRA activity both in a primary cell model system of latency and cells from aviremic individuals with HIV-1 treated with ART. Furthermore, GS143 reactivates latent HIV-1 without activating T cells, a desirable attribute for LRAs of clinical use. We showed that GS143 acts in a complementary fashion with at least two other classes of LRAs, thereby representing novel drug combinations for targeting HIV-1 latency. Finally, our results suggest that GS143 triggers a novel signaling pathway to reactivate latent HIV-1 that leads to the unconventional activation of NFκB p65, by initiating the noncanonical signaling via NIK, followed by activation of IKK leading to phosphorylation of p65 on S536 and its nuclear translocation. Moreover, we show that β-catenin inhibitors suppress reactivation HIV-1 by GS143, suggesting that β-catenin supports NF-κB output indirectly. Overall, our results suggest that the β-TrCP E3 ligase inhibitor GS143 represents a new type of LRA.

## Author summary

A major barrier to a cure for HIV-1 is the ability of the virus to hide for long periods of time in individuals on antiretroviral therapy. A possible strategy to get rid of latently infected cells is to find drugs that can turn on expression of HIV-1 proteins, leading to destruction of the infected cells by the virus or the immune system, while continued antiviral therapy would prevent new infections. To date, many compounds that can reverse HIV-1 latency lack a high degree of specificity. Compounds that could do so with a high degree of precision would be highly desirable for clinical use, as they would likely have fewer off-target effects and be less toxic than other LRAs. We found that GS143, an inhibitor of a specific protein degradation pathway (ubiquitin proteasome E3 ligase) strongly reactivates latent HIV-1 in primary cells and in patient samples without activating T cells. Furthermore, we show that GS143 reactivates latent HIV-1 via the unconventional activation of the NFκB signaling pathway. Thus, our study provides new insight into the function and mechanism of a new class of latency reversing agent and justifies investigation of GS143 as a possible candidate for further drug development.

## Introduction

A possible approach to eliminate the latent arm of HIV-1 infection involves the development of effective latency reversing agents (LRAs) [1–5]. Identification of LRAs that can activate latent virus with a higher degree of specificity than current LRAs is likely to produce fewer off-target effects and less toxicity than drugs lacking specificity.

Previously, we reported that the ubiquitin-proteasome system (UPS) is involved in the maintenance of HIV-1 latency, which presents the potential to develop more specific antagonists of HIV-1 latency [6]. The UPS plays an important role in gene expression, utilizing both its proteolytic and non-proteolytic activities [7]. Ubiquitin-dependent protein turnover is a highly regulated, multistep process. In general, proteins targeted for proteolysis by the multicomponent 26S proteasome, which is composed of two regulatory 19S subunits that abut a catalytic 20S core subunit, must be modified with at least a tetra-ubiquitin (Ub) chain. Generally, the first Ub is typically conjugated to a lysine on the target substrate, and the additional Ubs are attached at lysine 48 of Ub. Three proteins required for poly-Ub conjugation are E1 (Ub-activating enzyme), E2 (Ub-conjugating enzyme), and E3 (Ub ligase enzyme). In humans, there are at least 2 E1s, 35 E2s, and 600 E3s, and the combinatorial utilization of the three classes of protein confers a great deal of specificity to substrate ubiquitination and subsequent turnover [8,9]. Because of the great diversity of E3 ligases, they are the most important factor in the UPS for conferring specificity to substrate ubiquitination and subsequent turnover.

It was previously shown that signaling through the Wnt pathway can upregulate HIV-1 gene expression [10] and that the E3 ubiquitin ligase β-TrCP plays an important role in regulation through the Wnt pathway [11,12]. This prompted us to test if GS143, a small molecule inhibitor of β-TrCP that was previously developed as an inhibitor of NF-κB signaling, exhibited LRA activity [13]. We show that GS143 is a specific and potent LRA both in a primary cell model of latency and in cells from aviremic HIV-1-positive patients. Importantly, GS143 does not lead to T cell activation. Furthermore, GS143 acts in a complementary fashion with at least two different classes of LRAs, representing novel LRA combinations.

Many different factors including chromatin modification and binding of transcription factors such as NFкB, C/EBP, AP1 and Sp1 are important for HIV-1 transcription [14]. The full scope of signaling pathways involved in HIV-1 latency and reactivation awaits further research. Among the most potent LRAs identified to date protein kinase C (PKC) activators Bryostatin and Prostatin, function by activating the canonical NFкB pathway. Since NFкB is involved in inflammation and apoptosis, activation of this pathway is associated with more toxicity and tumorigenesis [15]. GS143 acts as an inhibitor of β-TrCP-mediated ubiquitination and proteosomal degradation of the NFкB inhibitor IкBɑ, by preventing its interaction with phosphorylated IкBɑ [13,16]. Recently, β-catenin, another target of β-TrCP has been shown to be a modulator of HIV-1 latency [17,18]. As IкBα and β-catenin both are potential targets of β-TrCP-mediated ubiquitination and degradation preceding the activation of these pathways, we examined these pathways to gain further insight into the mechanism of GS143-mediated HIV-1 reactivation.

## Results

### GS143 displays potent LRA activity in a primary cell model of HIV-1 latency

**Fig 1:**
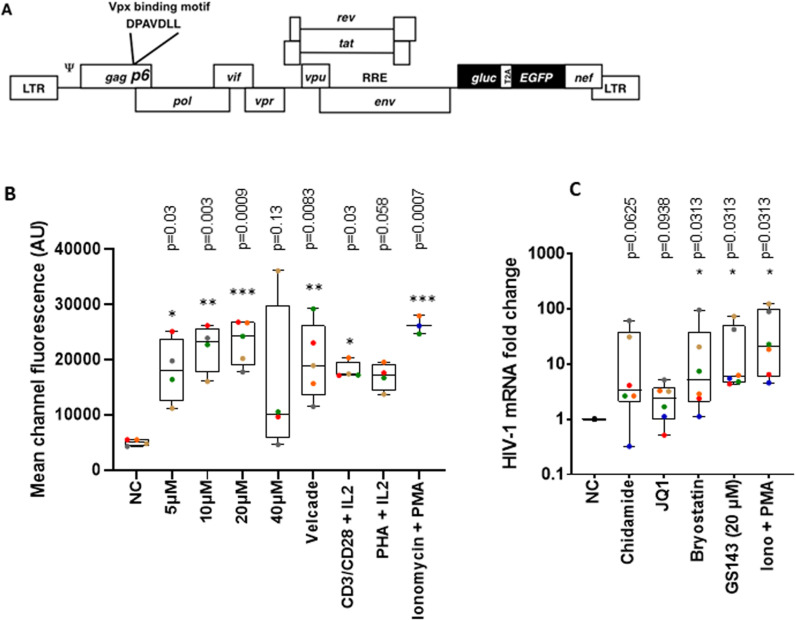
Activation of latent virus in the primary cell model by GS143 (A) Construct gGn-p6* is a replication competent vector with the Vpx binding motif (DPAVDLL) inserted within p6 as indicated. Abbreviations: *gluc* – *Gaussia* luciferase gene; T2A – T2A peptide promoting “ribosomal skipping”; *EGFP* – enhanced green fluorescent protein gene. (B) Resting CD4+ T cells infected with gGn-p6* were treated with indicated concentration of GS143, 10 nM Velcade, anti-CD3/CD28 beads plus 100 U/ml IL-2, 10 ng/ml phytohaemagglutinin (PHA) plus 100 U/ml IL-2, or 0.5 μM ionomycin plus 100 ng/ml phorbol 12-myristate 13-acetate (PMA) or with DMSO (NC negative control) in the presence of Raltegravir. GFP mean channel fluorescence from the infected cells was measured 48 hours later. Data from donor samples (n = 3 to 5) are presented as medians, interquartile ranges (IQR) and minimum and maximum with each donor represented by a dot are shown. Significance was determined by one-way ANOVA and Dunnett’s multiple comparisons comparing each treatment to untreated negative control (*p<0.05, **p<0.005 and ***p<0.0005). AU stands for arbitrary units. (C) HIV-1 mRNA expression in the resting CD4+ T cells (3~5 × 10^5^) from healthy donors (n = 6) was measured by RT-qPCR following treatment with Chidamide (20 µM), JQ1 (1µM), Bryostatin (10 nM), GS143 (20 µM), PMA (100 ng/ml) + Ionomycin (0.5 µM) [20] or DMSO (NC negative control) in the presence of Raltegravir. RNA measurements were normalized to the negative control. Each dot corresponds to a different donor and represents the average of two PCR reactions. Data are presented as medians, interquartile ranges (IQR) and minimum and maximum. Statistical significance was determined by non-parametric Wilcoxon matched-pairs signed rank test (two tailed). Statistically significant changes relative to NC were indicated as *P<0.05. In Fig 1B and 1C, each individual is represented by specific symbol in different treatments so that the dose (where applicable) and treatment effect can be followed for an individual donor.

A previously described primary model cell system was used for the initial testing of GS143 for LRA activity [19]. A replication-competent (RC) HIV-1 vector virus, gGn-p6* harboring a HIV-1 Vpx binding site within the p6 domain was used for infection (Fig 1A). This vector promotes more efficient infection of resting CD4+ T cells by allowing complementation with Vpx protein. To establish a latent virus infection, resting CD4+ T cells (CD4+CD25-CD69-HLA-DR- purity >99%) [19] were isolated from healthy donors and infected with Vpx-complemented gGn-p6* via spinoculation. This procedure typically results into the establishment of latent virus infection in 2 to 10% of the resting CD4+ T cells within 3 days (S1A Fig).

Latently infected cells were treated with 5 to 40 μM GS143 (structural formula shown in S1B Fig) for 48 hours followed by analysis of GFP marker expression by flow cytometry (Fig 1B). Mean channel fluorescence (MCF) measurements indicate that GS143 significantly upregulates GFP expression at concentrations ranging from 5 to 20 μM but not at 40 μM possibly due to toxicity at that concentration, suggesting that GS143 is a potent LRA, especially when compared to the positive controls including treatment with potent T cell activators such as PMA plus ionomycin, anti-CD3/anti-CD28 (aCD3/aCD28)-coated magnetic beads. GS143 also demonstrated comparable activity to proteasome inhibitor Velcade, previously demonstrated to be a robust LRA [5].

To measure the effect of GS143 on HIV-1 mRNA expression, a latent virus infection was established as above followed by treatment with the indicated LRAs (Fig 1C). HIV-1 mRNA was measured by RT-qPCR as previously described [20]. GS143 alone produced a potent signal comparable to the positive control PMA plus ionomycin, consistent with GFP expression (Fig 1B). Moreover, as shown in Fig 1C, the effect of GS143 on HIV-1 mRNA expression was comparable to that of Bryostatin-1, a PKC agonist [21,22] and compares favorably with well-known LRAs as JQ1 and Chidamide. Of note, here we used the previously described primer/probe set from 3’LTR sequences [20] detecting only correctly terminated poly A containing transcripts excluding the premature read through transcripts. These full-length correctly terminated transcripts are likely to produce replication competent virus. This assay is more stringent than commonly used measurement of RNA using primers containing HIV-1 gag sequences.

### GS143 treatment does not induce T-cell activation or significant cytotoxicity

**Fig 2:**
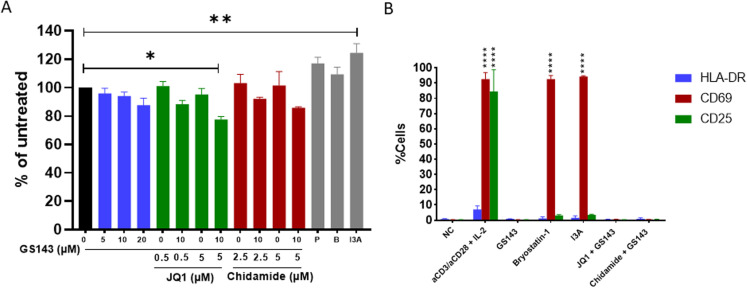
Toxicity and activation profile of GS143 alone and in combination with other LRAs. Primary resting CD4+ T cells (2x10^5^) from three healthy donors (n=3) were treated with (A) GS143 alone (0, 5, 10 and 20 µM) or in combination with JQ1 (0.5 and 5 µM) or Chidamide (2.5 and 5 µM) or with indicated compounds. After 48 hours, cells were (A) assayed with the CellTiter-Glo Luminescent Cell Viability Assay or (B) incubated with fluorescently tagged antibodies specific to the indicated T-cell activation markers and analyzed by flow cytometry as described in methods. The values are displayed as a percentage of the untreated negative control (100%) ± SEM. Significance was determined by one-way ANOVA and Dunnett’s multiple comparisons comparing each treatment to the untreated negative control (*p<0.05, **p<0.005) P, Prostratin; B, Bryostatin-1; I3A, Ingenol-3-angelate (A). Mean ± SEM and significance determined by one-way ANOVA and Dunnett’s multiple comparisons test within each measurement (HLA-DR, CD69 and CD25) compared to untreated negative control are shown (****p<0.0001). aCD3/aCD28, beads coated with antibodies against CD3/CD28; I3A, Ingenol-3-angelate; NC, untreated negative control (B).

To determine the safety of GS143, we examined whether GS143 induces cytotoxicity or T cell activation. Uninfected resting CD4+ T cells from healthy donors were incubated with the indicated compounds for 48 hours. Cytotoxicity was determined using the CellTiter-Glo Luminescent Cell Viability Assay (see Methods), which measures the cell number as a function of cellular ATP. As seen in Fig 2A, GS143 did not exhibit significant cytotoxicity at any of the concentrations examined. Similar results were seen for previously characterized LRAs JQ1 and Chidamide [19]. However, combinations of 5 µM JQ1 and 10 µM GS143 showed statistically significant increase in cytotoxicity (Fig 2A). Prostratin, Bryostatin-1, and Ingenol-3-angelate (I3A), which are PKC agonist LRAs did not show any significant cytotoxicity compared to the untreated control. However, I3A showed increase in cell proliferation compared to the control untreated cells.

To ensure that the LRA activity exhibited by GS143 was not due to an indirect effect of T cell activation, T cell activation profile was examined. Resting CD4+ T cells from healthy donors following treatment with GS143 alone or in combination with JQ1 or Chidamide were examined for the expression of T cell activation markers HLA-DR, CD69, and CD25 (Fig 2B). As a positive control, fully activated cells treated with magnetic beads coated with antibodies to CD3 and CD28, along with IL-2 was used. Notably, neither GS143 (10 µM) alone or in combination with JQ1 or Chidamide significantly increased the expression of HLA-DR, CD69, or CD25 indicating little or no T cell activation. On the contrary, following treatment with PKC agonists bryostatin-1 and I3A, a dramatic increase in CD69 expression was observed (Fig 2B). These observations indicate that PKC agonists induce partial T-cell activation, consistent with increased cellular proliferation seen at least in I3A in Fig 2A. Neither of these properties are desirable in LRAs for clinical use

### GS143 induces HIV-1 mRNA expression in resting CD4+ T cells isolated from individuals with HIV-1

**Fig 3:**
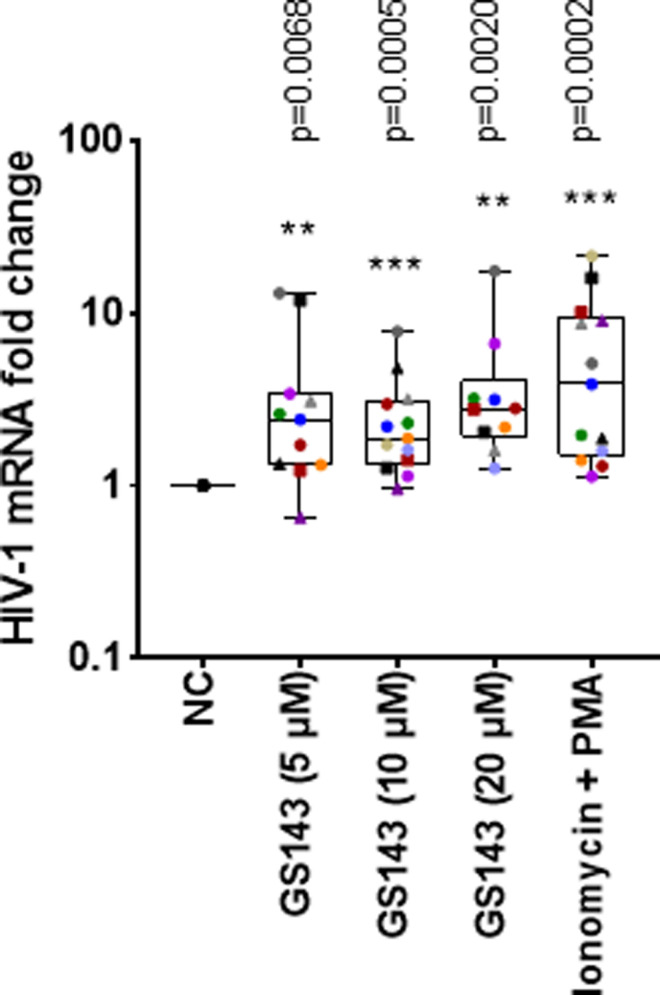
HIV-1 expression induced by GS143 in resting CD4 **+** **T cells isolated from individuals with HIV-1 on suppressive ART.** Resting CD4+ T cells (2.5-5 x 10^6^) were isolated from the blood of individuals living with HIV-1 (n = 10 -13) and treated with GS143 or ionomycin plus PMA or left untreated for 24 hours. Total RNA was isolated, and HIV-1 mRNA was quantified by RT-qPCR as previously described [20]. Signals were normalized to the mock treated negative control (NC). Each dot corresponds to a different subject and represents the average of two PCR reactions. Data are presented as medians, interquartile ranges (IQR) and minimum and maximum. Statistical significances relative to untreated negative control (NC) were determined by Wilcoxon matched pairs signed rank test (two tailed) assuming non-parametric distribution and indicated as **P < 0.005, ***P < 0.0005. Each individual is represented by specific symbol in different treatments so that the dose (where applicable) and treatment effect can be followed for an individual donor.

Blood samples were obtained from consented individuals with HIV-1 undergoing antiretroviral therapy with CD4+ T cell counts greater than 350 per μl and viral loads below 50 copies per ml for at least 5 years and many for over 10 years. Resting CD4+ T cells were isolated from subjects’ blood, followed by treatment with GS143 for 24 hours after which mRNA was isolated and HIV-1 mRNA quantified by RT-qPCR [20]. Treatment with GS143 resulted in a significant increase in viral RNA levels at all three concentrations tested as seen in positive control PMA plus ionomycin (Fig 3). We did not observe any dose response of GS143 on HIV-1 reactivation. As expected, a significant variation from patient to patient was observed by us and others when testing other LRAs utilizing patient samples [23,24]. These data indicate that, consistent with the observation in primary cell model (Fig 1A and 1B), GS143 treatment causes reactivation of latent HIV-1 in resting CD4+ T cells from patients undergoing long-term antiviral therapy. Using this assay, baseline intracellular HIV-1 mRNA could be detected in samples from individuals with HIV-1 by Bullen *et al* [20]. Furthermore, the primer/probe set used here offers much more stringent determination than commonly used measurement of RNA with primers using HIV-1 gag sequences.

### GS143 reactivates latent HIV-1 in combination with previously identified LRAs

**Fig 4:**
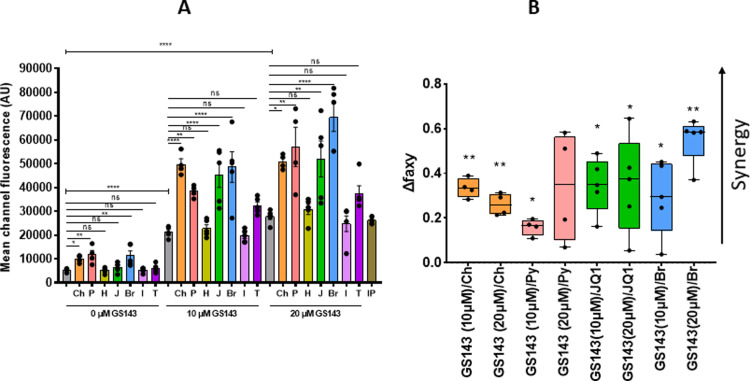
GS143 combinational drug assay on HIV-1 activation in resting CD4 **+** **T cell.** Resting CD4+ T cells from healthy donors infected with gGn-p6* were treated with the indicated concentrations of GS143, alone or in combination with LRAs. Ch, 20 μM Chidamide; P, 20 μM Pyroxamide; H, 1 mM Hexamethylene bisacetamide (HMBA); J, 1 μM JQ1; B, 10 nM Bryostatin-1; I, 0.5 μM Ionomycin; T, 20 ng/ml TNFα, and IP, 0.5 μM Ionomycin plus 100 ng/ml PMA in the presence of Raltegravir. (A) GFP mean channel fluorescence from the infected cells was measured 48 hours later. Columns represent mean ± SEM from healthy donor samples (n = 3-5) each indicated with dots. Statistical significances within each group derived by one-way ANOVA and Dunnett’s multiple comparisons test within each measurement (0, 10 and 20 µM GS143) are indicated as *P < 0.05, **P < 0.005, ***P < 0.0005 and ****P < 0.0001. Gray bars represent the untreated negative control or treatment with GS143 alone at the indicated concentrations. AU, arbitrary unit. (B) Bliss independence analysis was performed to examine the synergistic effect of GS143 in combination with Chidamide, Pyroxamide, JQ1 and Bryostatin (statistically significant drug combinations relative to GS143 shown in A) compared to a single LRA. *p < 0.05 and **p < 0.001 compared between faxyO and faxyP by two-tailed paired t-tests (Methods).

Since LRA combinations rather than any single LRA may generate sufficient efficacy to reactivate latent HIV-1 [23–25], GS143 was tested in combination with previously described LRAs. Latently infected resting CD4+ T cells were treated with the indicated compounds alone or in combination with GS143 for 48 hours and the mean channel fluorescence was measured by flow cytometry (Fig 4A). Four out of seven combinations including well known LRAs JQ, Bryostatin-1 [23,26] and histone deacetylase inhibitors (HDACis) Chidamide and Pyroxamide displayed significant increase of MCF compared to the individual LRAs. It is noteworthy that these HDACis contain a benzamide functional group and a pyridyl cap, which we previously showed were the most active LRAs within the HDACi class and with the least pronounced toxicity [19]. On the other hand, hexamethylene bisacetamide (HMBA), which releases P-TEFb from 7SK snRNA [27], or Ionomycin exhibited no significant effect, as previously shown in several primary cell latency models [28,29]. In addition, HMBA and Ionomycin in combination with GS143 did not show any increase in MCF compared to GS143 alone. TNFα alone or in combination with GS143 demonstrated no significant activation, as reported before [29].

The synergy of GS143 with JQ1, Bryostatin, Chidamide and Pyroxamide was assessed by Bliss independence model for combined drug effect [30]. These LRAs in combination with GS143 resulted in a significant increase in experimentally observed effects (faxyO) compared with the effects predicted (faxyP) by the Bliss model (see Methods) as shown in Fig 4B. Thus, these drug combinations were found to exhibit synergy.

### GS143-mediated HIV-1 reactivation involves NFкB and β-catenin pathways

The basal transcription of the HIV-1 promoter (5’ LTR) is regulated by numerous host cell transcription factors and their associated cofactors. Among them the HIV-1 5’ LTR contains two copies of the NFкB consensus binding site, which binds to RELA:p50 or RELB:p52 dimers depending on the activation of canonical or non-canonical NF-кB pathways, respectively [31–33]. In addition, the HIV-1 5’ LTR harbors three consensus binding sites for LEF1 [34,35], a member of LEF-1/TCF family and the downstream effector of the Wnt/β-catenin signaling pathway.

#### Accumulation of IкBɑ and β-catenin.

While GS143 specifically blocks interactions between β-TrCP and phosphorylated IкBɑ, inhibiting its ubiquitation and degradation [13] and Erioflorin, another β-TrCP E3 ligase inhibitor also inhibits degradation of β-TrCP targets including IкBɑ and β-catenin [36]. Given the importance of the NF-кB and β-catenin pathways in HIV-1 reactivation, we first examined whether GS143 prevents β-TrCP-mediated degradation of IкBɑ and β-catenin.

**Fig 5:**
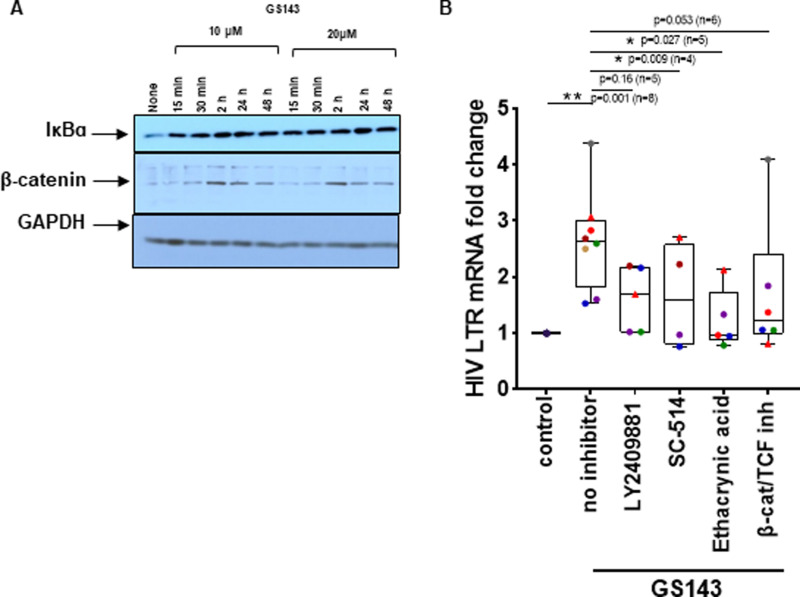
GS143 increases IкBɑ and **β-catenin accumulation in resting CD4+** **T cells.** Primary resting CD4+ T cells (1-2 × 10^6^) isolated from healthy donors were treated with 10 or 20 µM of GS143 for the indicted time or left untreated and whole cell lysates were analyzed by Western blotting. (A) Kinetics of IκBα and β-catenin accumulation after GS143 treatment was examined by immunoblotting with IкBɑ, β-catenin and GAPDH antibodies. A representative of 3-6 independent experiments using different subjects is shown. Quantification of bands is shown in S4 Fig. (B) Resting CD4+ T cells (n = 4-8) were infected with gGn-p6* and latently infected cells were treated with NFкB or β-catenin inhibitors followed by treatment with GS143 in the presence of Raltegravir. After 48 h, total RNA was extracted and analyzed by RT-qPCR. RNA measurements were normalized to the negative control. Each dot represents a different donor. Data are presented as medians, interquartile ranges, minimum and maximum. Statistical significances relative to untreated control determined by Wilcoxon matched pairs signed rank test (two tailed) assuming non-parametric distribution indicated as *P < 0.05 and **P < 0.005 compared to the control. P values and number of donors per comparison is shown.

Analysis of whole cell lysates following treatment of resting CD4+ T cells with GS143 for different durations showed accumulation of IкBɑ and β-catenin compared to the untreated control (Fig 5A). Furthermore, NFкB pathway inhibitor SC514 and β-catenin pathway inhibitor ethacrynic acid [37] significantly inhibited GS143-mediated HIV-1 LTR mRNA expression in latently infected resting CD4+ T cells (Fig 5B) indicating the involvement of these pathways in the reactivation of the latent HIV-1.

#### cIAP2 downregulation, NIK stabilization and IKKɑ/β phosphorylation.

**Fig 6:**
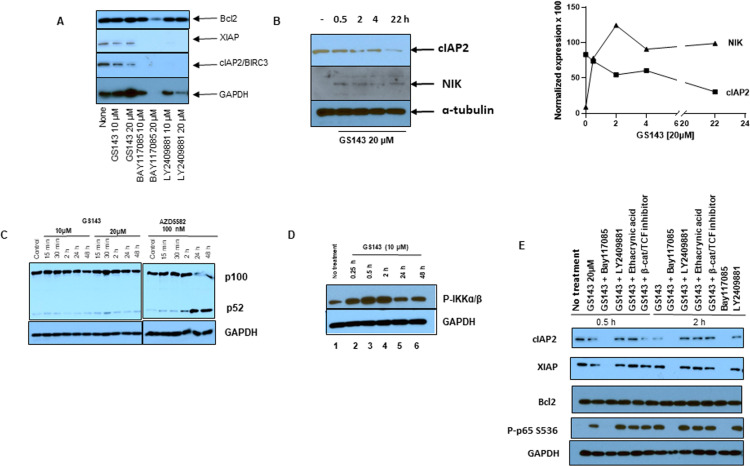
Downregulation of cIAP2, stabilization of NIK, phosphorylation of IKK **α/β** **and p65.** Primary resting CD4+ T cells (1-2 × 10^6^) isolated from healthy donors were treated with 10 or 20 µM of GS143 for the indicted time or left untreated and whole cell lysates were analyzed by Western blotting. (A) Expression of survival genes Bcl2, XIAP, cIAP2 examined in lysates prepared 24 h after GS143 treatment by immunoblotting, (B) Kinetics of cIAP2 downregulation and NIK stabilization, and (C) Kinetics of p100 and p52 expression were examined by immunoblotting with appropriate antibodies. SMAC mimetic AZD5582 was used as a positive control of p100 processing. (D) phosphorylation of IKK were analyzed in lysates treated with GS143 for the indicated time. (E) Primary resting CD4+ T cells isolated from healthy donors were treated with 20 µM of GS143 with or without 30 min pretreatment with NFκB or β-catenin inhibitors for the indicated time or left untreated. Whole cell lysates were analyzed by immunoblotting with cIAP2, XIAP, Bcl2, P-S536-p65 and GAPDH. Experiments were repeated with three different donors. Quantification of cIAP2, NIK, P-IKKɑ/β and P-S536-p65 bands is shown in S5 Fig.

Since GS143 inhibits IкBɑ degradation required for the activation of the canonical NF-кB pathway, we examined whether GS143 activates the non-canonical NF-кB signaling pathway, as previously seen with SMAC-mimetics. Treatment with SMAC mimetics leads to degradation of cIAP1 followed by stabilization of NIK and downstream activation of the non-canonical pathway [32,38]. Since the basal NF-кB activity is required for maintaining the anti-apoptotic family of proteins such as Bcl2 and inhibitor of apoptosis proteins (IAP) including cIAP2 and XIAP in resting T cells [39], we examined the effect of GS143 on their expression. As expected, NF-кB pathway inhibitors BAY117085 and LY2409881 reduced the level of both cIAP2 and XIAP (Fig 6A). The higher doses (20 µM) of these inhibitors caused toxicity and as a result GAPDH was not detected. We found that GS143 treatment reduced the level of cIAP2 but not Bcl2 or XIAP. Furthermore, we observed that downregulation of cIAP2 in GS143 treated cells (Fig 6B) correlated with the accumulation of NIK. This is reminiscent of the activation of the noncanonical NFκB signaling pathway by SMAC mimetics such as AZD5582, which is known to reactivate HIV-1-infected cells by activating the non-canonical NFкB signaling [38]. However, in contrast to SMAC mimetic AZD5582, GS143 treatment led to little or no processing of p100 to p52 compared to control untreated cells (Fig 6C). This is most likely due to GS143-mediated inhibition of β-TrCP-dependent processing of p100 to p52.

IкB kinase (IKK) complex, the master kinase regulating the activation of the NF-кB pathway comprises of two kinase (IKKɑ and IKKβ) and a regulatory (IKKɣ) subunit [40]. Given the role of NIK in the activation of both canonical and noncanonical NF-кB signaling [41,42], we examined whether upregulation of NIK (Fig 6B) correlate with the activation/phosphorylation of IKKɑ and IKKβ in GS143 treated resting CD4+ T cell. GS143 treatment led to a rapid increase in the phosphorylation of IKKɑ/β visible as early as 15 minutes (Fig 6D). Taken together, these results provide a potential pathway for GS143-mediated IKK activation in resting CD4+ T cells in the absence of receptor mediated signaling.

#### Downstream phosphorylation of NFκB transcription factor p65/RelA.

p65 is a key transcription factor activated by the canonical NFκB pathway. The phosphorylation of p65 at different serine residues induces conformational changes impacting its ubiquitination, stability and protein-protein interactions [43,44]. In particular, it has been shown that phosphorylation of p65 at S536 located in its C-terminal transcriptional activation domain (TAD) reduces its binding affinity to IкBɑ as well as p50 and promotes its nuclear translocation, which subsequently leads to the transcriptional activation of the target genes [16,45]. To determine whether the observed activation of IKK in GS143 treated cells is accompanied by the phosphorylation of p65, we examined the level of P-S536 p65 in resting CD4+ T cells. As shown in Fig 6E, GS143 treatment dramatically increased the level of phosphorylated P-S536 p65 in resting CD4+ T cells. As expected, P-S536-p65 levels were not reduced by β-catenin inhibitors and reduced by NFκB pathway inhibitor BAY117085. However, IKK2 inhibitor LY2409881 did not reduce p65 phosphorylation suggesting the possibility of involvement of kinases other than IKK2 in p65 phosphorylation.

#### Protein-protein interaction and nuclear translocation.

**Fig 7:**
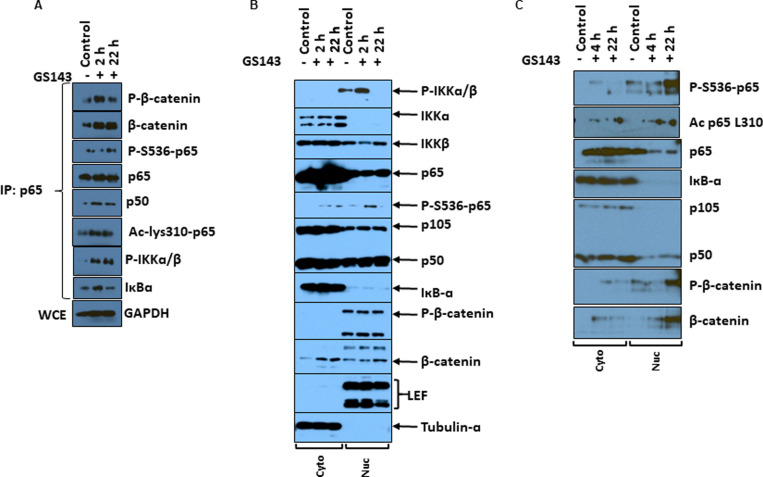
Analysis of nuclear localization and interaction of p65 with other proteins. (A) whole cell lysates were prepared from resting CD4+ T cells (n = 3) following 2 and 22 h treatment with GS143 or left untreated. Immunoprecipitation was performed with 200 µg of extracts (Methods) with p65 antibody. Immune complexes were analyzed with immunoblotting with specific antibodies. (B) Cytoplasmic and nuclear extracts were prepared from resting CD4+ T cells (n = 5) treated with GS143 for 2 and 22 hours or left untreated (Control) and analyzed by western blotting. Tubulin-α and LEF-1 served as cytoplasmic and nuclear markers, respectively. Quantification of P-S536-p65 is shown in S5 Fig. (C) Cytoplasmic and nuclear extracts were prepared from long-term culture of PHA-blasts (n = 6) expanded with IL-2 following 4 and 24 h treatment with GS143 or left untreated. Immunoprecipitation was performed with 100-200 µg of extracts (Methods) with p65 antibody. Immune complexes were analyzed with immunoblotting with specific antibodies.

Given that GS143 induced p65 phosphorylation, we set out to identify key signaling protein interactions with p65 relevant to the activation of HIV-1. Immunoprecipitation assays with anti-p65 were performed using whole cell lysates from GS143 treated and untreated control resting CD4+ T cells. Interestingly, both phosphorylated β-catenin and P-IKKɑ/β coimmunoprecipitated with p65. Although β-catenin was shown to complex with p65, no association of LEF1 or TCF1 was detected in the complex. Since the resting CD4+ T cells express LEF1 and TCF1 but not TCF3 or TCF4 (S2A Fig), we examined for the presence of LEF1 and TCF1 in the immune complex. Phosphorylation of p65 at serine 536 and acetylation of p65 increased with treatment with GS143 (Fig 7A). Acetylation of p65 requires interaction with coactivator proteins CBP/p300. However, CBP/p300 did not coimmunoprecipitate with p65.

Next to examine whether P-S536 p65 and P-β-catenin translocate to the nucleus, cytoplasmic and nuclear extracts were prepared from resting CD4+ T cells treated with GS143. As expected IкBɑ was detected predominantly in the cytoplasm and p50 was detected both in cytoplasm and nucleus while more p105 was detected in cytoplasm (Fig 7B). Interestingly, P-S536 p65 was detected both in the cytoplasm and in the nucleus while phosphorylated β-catenin was detected only in the nucleus (Fig 7B).

Finally, to determine whether β-catenin interacts with p65 in the nucleus, cytoplasmic and nuclear extracts from GS143 treated and untreated control were each subjected to immunoprecipitation with anti-p65. Since these assays require much more protein than immunoblotting, long-term cultures of PHA-treated T cells derived from PBMC (Methods) as described previously [46] were used. Clearly, GS143 led to a significant increase in nuclear phospho-p65 (ser536) protein complex (Fig 7C) indicating that a greater proportion of nuclear p65 is phosphorylated at S536. Note that the amount of p65 is significantly less in the nucleus than in cytoplasm. Furthermore, very little or no association of IкBɑ with p65 was detected in the nucleus. p65-p50 complex was more abundant in the cytoplasm than in the nucleus (Fig 7C). Interestingly, the association of P-β-catenin with p65 was observed in the nucleus in GS143 treated cells. Nuclear β-catenin might participate in HIV-1 transcription directly via LEF binding or indirectly by interacting with nuclear cofactors CBP and p300 facilitating acetylation of p65 required for transactivation [47].

**Fig 8:**
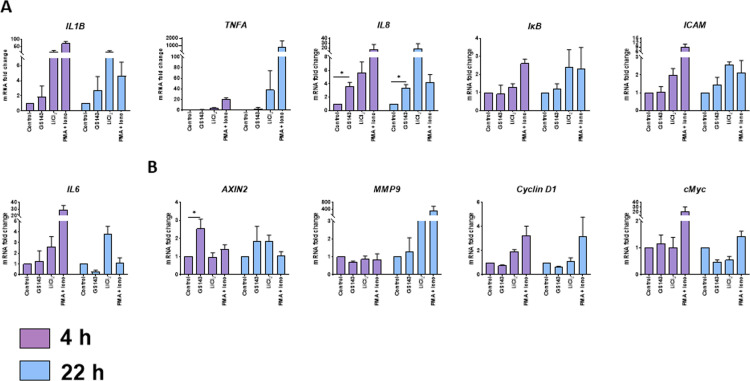
Analysis of NF-кB (A) and **β-catenin (B) target genes.** Long-term cultures of PHA-treated T lymphocytes (n = 3) were treated with GS143 (10 µM), LiCl2, PMA + Ionomycin or left untreated for 4 or 24 h. RNA was analyzed by RT-qPCR using SYBR green. Statistical significances of GS143 mediated *IL8* and *AXIN2* expression was determined with two-tailed t-test assuming equal variance.

To further substantiate the activation of the NFκB and β-catenin pathways by GS143, we examined the effect of GS143 on downstream target genes of these signaling pathways. NFкB and β-catenin target genes *IL8* and *AXIN2*, respectively, were induced by GS143 (Fig 8). In contrast, no effect was observed on the activation of the other targets examined. Upregulation of *IL8* mRNA is consistent with the previous finding that p65 phosphorylated at S536 is not regulated by IкBɑ and activates distinct target genes such as *IL8* [43]. Taken together, these data provide a plausible pathway by which GS143 may reactivate latent HIV-1, involving β-catenin and nonconventional activation of NFκB, with limited off target gene activation.

## Discussion

The UPS utilizes different combinations of E1, E2, and E3 ligases to confer a high degree of specificity on numerous specific UPS pathways [48]. Thus, the existence of more than 600 E3 ligases presents the possibility of developing highly specific LRAs that target an individual E3 ligase involved in maintaining HIV-1 latency. Unlike proteasome inhibitors, which exhibit HIV-1 LRA activity [6] with relatively broad effects upon inhibition of the UPS, specific inhibitors of E3 ligases are expected to show remarkable specificity. Using both a primary cell model of latency and blood specimens from individuals with HIV-1- without viraemia, here we demonstrated that E3 ligase β-TrCP inhibitor GS143 is a strong LRA, which functions without activating T cells as indicated by no alteration of the expression of T-cell activation markers HLA-DR, CD69, and CD25 in resting CD4+ T cells (Fig 2B). Furthermore, GS143 shows synergy with other classes of LRAs such as Chidamide, Pyroxamide, JQ1 and Bryostatin (Fig 4A and 4B). Also, GS143 treatment does not alter the expression of CD4 (S2B Fig). In infected cells viral protein vpu interacts with CD4 and recruites β-TrCP leading to its degradation [49]. Thus, GS143 is not likely to affect vpu function requiring vpu-CD4 interaction in infected cells.

Yukl *et al.* [50] reported that the HIV-1 latency in CD4+ T cells from people with HIV-1 often involves blocks in elongation, termination/polyadenylation and multiple splicing. Further, the existence of multiply spiced RNA (msRNA) is more predictive of replication competent virus production after HIV-1 latency reversal [51–53]. Of note, the primer/probe set used in this study is in the 3’LTR and contains sequences complementary to the poly A tail. Thus, this primer/probe set detects all fully elongated and correctly terminated poly A containing transcripts (unspliced, single splice and multiple splice) excluding the premature read through transcripts potentially excluding defective and replication incompetent virus. The primer/probe set used here detects both unspliced as well as spliced viral RNA. A limitation of this study is that it relies solely on readouts of viral RNA and flow cytometry-based EGFP reporter protein expression. Although RNA-based read outs were used by others (for example [54]), it remains to be determined if GS143 can also induce production of replication competent virus. It has been reported that transcriptionally competent viral reservoirs may not generate replication competent virus due to post transcriptional block but still can trigger innate antiviral responses. This may effectively help eliminate virus infected cells. Thus, RNA based assays can provide important measures of LRA activity [55].

Eradication of latent reservoirs of HIV-1 seeded early in the infection either by the direct infection of resting CD4+ T cells or reversal of infected activated CD4+ T cells into a quiescent state is critical for the cure of HIV-1 [56,57]. Establishment and maintenance of HIV-1 transcriptional silencing is complex and regulated by multiple cellular factors ranging from host transcription factors to the chromatin microenvironment in quiescent T cells. NFκB plays a complex role in the establishment and reversal of latency in memory T cells, the primary reservoir of the latent HIV-1 provirus. NFκB dependent suboptimal expression of pro-survival genes are essential in maintaining the latent state. On the other hand, stronger NFκB signaling is required for latency reversal [58]. GS143, which interferes with the degradation of IκBɑ by β-TrCP is expected to inhibit canonical NFκB signaling. Given the pivotal role of NFκB in the maintenance and reversal of HIV-1 latency, it may seem paradoxical at first that GS143, a known inhibitor of NFκB pathway activates HIV-1 transcription both in the latently infected cell model (Fig 1A and 1B) and in cells from ART suppressed patients (Fig 3).

It is known that basal NFκB signaling is required in the resting CD4+ T cells for low level of transcription of the genes required for survival, including cIAPs [39]. Consistent with this, we observed that treatment with GS143 leads to downregulation of cIAP2 in resting CD4+ T cells (Fig 6A, 6B and 6E). Consistent with the reduced level of cIAP2, we observed NIK stabilization in GS143-treated resting CD4+ T cells (Fig 6B). It has been shown that the noncanonical NFκB signaling pathway is regulated through the constitutive cIAP1, cIAP2, TRAF2 and TRAF3-mediated degradation of the kinase NIK in unstimulated cells. Activation of specific tumor necrosis factor receptors such as CD40 promotes ubiquitination and degradation of TRAF3 by cIAP1 and cIAP2 and subsequently, leads to the stabilization and accumulation of NIK. This results in the activation of IKKɑ, leading to β-TrCP-mediated proteolytic processing of p100 to p52. p52-Rel B complex translocates to the nucleus leading to DNA binding and subsequently, target gene expression [59]. Our results indicate that GS143 initiate the activation of the noncanonical NF-κB signaling pathway through stabilization of NIK and phosphorylation of IKK.

SMAC mimetic compounds like AZD5582 [38] and SBI-6037142 [32] are shown to promote latency reversal by activating noncanonical NFκB signaling initiated by the downregulation of cIAP1 and culminating into β-TrCP-mediated p100 processing and nuclear migration of p52:RELB. Both SMAC mimetics and GS143 reactivate latent HIV-1 and both initiate noncanonical NFκB signaling. However, they function in different ways. Unlike SMAC mimetics, we observed that GS143 inhibits β-TrCP-mediated processing of p100 (Fig 6C), which is consistent with ubiquitination site (K856) located upstream of the phosphorylation site of p100 is analogous to the ubiquitination site (K22) of IκBα [59]. While GS143 prevents β-TrCP-mediated processing of p100 and thus further downstream signaling via the non-canonical NFκB signaling pathway, we observed phosphorylation of IKKα/β in GS143 treated resting CD4+ T cells (Fig 6D) and signaling downstream of IKK. This is consistent with the ability of NIK to phosphorylate both IKKα and IKKβ and further, regulate both the canonical and noncanonical NFκB signaling pathways [41,42]. Interestingly, we also observed an increased p65 phosphorylation on S536 in GS143 treated resting CD4+ T-cells. It is worth noting that the phosphorylation of p65 on S536 located in the transactivation domain facilitates its nuclear localization, DNA binding and subsequently target gene expression [43]. In agreement with this, a major portion of nuclear p65 was phosphorylated at S536 (Fig 7C) and was not associated with IκBα. Our results therefore indicate that GS143 leads the unconventional activation of NFκB p65 by initiating the noncanonical signaling via NIK, followed by activation of IKK leading to phosphorylation of p65 on S536.

Some genes are transcribed selectively by NFκB complex containing a form of p65 phosphorylated on serine 536 [45]. For instance, P-S536 p65 binds to the promoters of distinct subset of genes like *IL8* and activates their transcription [60,61]. Here we assessed the expression of several downstream target genes of NFκB in CD4+ T cells and found that only *IL8* was induced by GS143 (Fig 8), a finding consistent with the activation of P-S536 p65 by GS143. Therefore, GS143 triggers an alternate pathway of NFκB p65 activation without involving IκBɑ degradation via βTrCP.

In addition to accumulation of IκBɑ, we also observed accumulation of β-catenin in cells treated with GS143 (Fig 5A). Normally, in the unstimulated cells in the absence of Wnt signaling, β-catenin is constantly phosphorylated at Ser33, Ser37 and Thr41 and subsequently undergoes β-TrCP-mediated degradation resulting into low level of β-catenin in the cytoplasm and the nucleus. In response to Wnt ligand, phosphorylation of β-catenin is suppressed and unphosphorylated β-catenin is stabilized and translocated to the nucleus and subsequently binds to LEF/TCF to activate target genes [62]. Interestingly, we show that phosphorylated-β-catenin is primarily nuclear (Fig 7B) in resting CD4+ T cells treated with GS143. In addition, β-catenin was coprecipitated by p65 indicating that β-catenin is in complex with p65 (Fig 7A and 7C). Consistent with our observation that inhibitors of β-catenin suppress the activation of HIV-1 by GS143 in resting CD4+ cells (Fig 5B), these results further suggest a role for β-catenin in GS143-mediated HIV-1 reactivation. However, it remains to be seen whether this involves a direct engagement of β-catenin/LEF-1/TCF complex binding on HIV-1 LTR leading to activation the signaling pathway or via an indirect modulation of NFκB function by β-catenin in GS143 treated cells. The fact that only *AXIN2* but none of the other β-catenin target genes that we examined (*MMP9*, *Cyclin D1* or *cMyc*) was induced by GS143 in resting CD4+ T cells (Fig 8B) suggest the absence of Wnt signaling in this context. Thus, it appears that in GS143 treated resting CD4+ T cells NFκB and β-catenin operates on very specific proteins without wide-spread gene activation (Fig 8). We hypothesize that the role of β-catenin in GS143 mediated HIV-1 reactivation may be supporting NFκB activation. Indeed, β-catenin has been shown to positively regulate NFκB function by modulating the interaction of CBP or p300 with p65 leading to the acetylation required for the trans activation of p65 [47], an effect independent of Wnt signaling.

Both positive and negative regulation of HIV-1 reactivation by β-catenin has been reported [17,18]. A recent report [17] shows that the inhibition of Wnt signaling by specific inhibitors leads to the activation of HIV-1 transcription via the TCF4 binding site located on HIV-1 LTR upstream of binding sites of NFкB and AP1 [17]. They further showed that β-catenin pathway inhibition by adavivint, which inhibits Wnt signaling and decreases β-catenin enhances latency reversal by a variety of other LRAs. They proposed that β-catenin positively regulates both TCF4 and c-myc, which impairs NFκB binding and recruits HDAC to HIV-1 LTR, respectively and inhibits transcriptional activation of HIV-1 [17,63]. On the contrary, we observed an inhibition of HIV-1 reactivation in GS143 treated CD4+ T cells by ethacrynic acid [37], which interferes with the association β-catenin with LEF-1 suggesting the importance of β-catenin-LEF-1 interaction in GS143-mediated HIV-1 reactivation (Fig 5B). It is worth noting that TCF1 and LEF1 but not TCF4 were readily detected in resting CD4+ T cells (S2A Fig). However, we did not observe transcriptional activation of β-catenin downstream target genes such as *cMyc* by GS143 in resting CD4+ T cells (Fig 8) as proposed [17]. Therefore, it appears that the involvement of β-catenin in HIV-1 reactivation is necessary in GS143 treated cells but independent of Wnt signaling. Coimmunoprecipitation of β-catenin and p65 suggest that β-catenin is a part of NFκB transcription complex and possibly enhance NFκB-mediated HIV-1 transcription in a Wnt independent manner.

**Fig 9:**
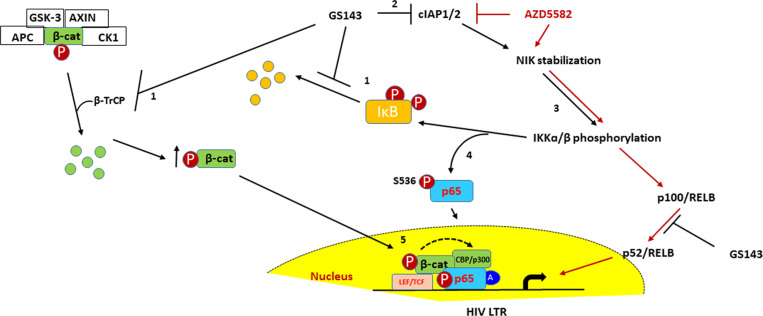
A model proposed for GS143-mediated HIV-1 reactivation. GS143 treatment results into accumulation of (1) IκBα and β-catenin by inhibition of their interaction with β-TrCP. [2] Inhibition of basal NFкB signaling leads to the downregulation of cIAP2, which in turn leads to the stabilization of NFкB inducing Kinase (NIK) and phosphorylation IкB kinases (IKK). [3] NIK phosphorylates and activates IKKɑ/β. Thus, GS143 partially activates non-canonical pathway that does not culminate into the processing of p100 leading to the release of the p52 and subsequently nuclear translocation of p52-RelB. GS143 interferes with β-TrCP-mediated processing of p100. [4] Activation of IKK leads to phosphorylation of p65 at serine 536 located in the transactivation domain. This modification is shown to reduce the affinity of p65 for IкBɑ and p50 and promote its nuclear translocation and DNA binding that could lead to HIV-1 reactivation. [5] β-catenin supports p65 output in the nucleus possibly by promoting acetylation of p65. SMAC-mimetic AZD5582-mediated activation of non-canonical NFκB signaling pathway is also shown.

Based on all these observations, we propose a hypothetical model for GS143-mediated HIV-1 reactivation (Fig 9) involving the activation of an alternate route for NF-кB signaling as well as β-catenin. In summary, GS143 represents a new class of HIV-1 latency antagonist that acts by inhibiting a specific E3 ligase, β-TrCP. These results support the concept that modulating a specific UPS pathway involved in regulating HIV-1 latency can produce not only a potent LRA, but one with potentially less toxic side effects due to greater specificity. As this study is done *ex vivo* with primary cells, it is technically challenging to provide direct proof of this model at present and is beyond the scope of this study. GS143 exhibits synergy with other classes of LRAs such as JQ1, Bryostatin-1, Pyroxamide and Chidamide in the latency model system (Fig 4). As our laboratory and others previously proposed, the use of LRA combinations rather than any single LRA may be necessary to achieve higher efficacy to produce sufficient latent virus clearance to eradicate the latent reservoirs. Therefore, additional research exploring GS143 as a possible candidate for further drug development to eliminate cells with latent HIV-1 is warranted.

## Methods

### Ethics statement

This study involving blood collection from individuals with HIV-1 was reviewed and approved by the Institutional Review Board of Rutgers University under protocol Pro20160001316. All study participants were adults and provided written informed consent. All samples were anonymized. PBMCs were isolated from deidentified healthy donor leukocytes purchased from the New York Blood Center.

#### Culture media.

All non-adherent cells were cultured in RPMI 1640 GlutaMAX, HEPES medium (Thermo Fisher Scientific) supplemented with 10% BioTC-Fetal Bovine Serum, (LDP), MEM Non-essential amino acids solution (Thermo Fisher Scientific), and 100 U/ml penicillin-100 μg/ml streptomycin solution (Thermo Fisher Scientific). Adherent cells were cultured in DMEM GlutaMAX medium (Thermo Fisher Scientific) supplemented with 10% BioTC-Fetal Bovine Serum, (LDP), MEM Non-essential amino acids solution (Thermo Fisher Scientific), and 100 U/ml penicillin plus 100 μg/ml streptomycin solution (Thermo Fisher Scientific).

#### Reagents.

The following reagents were used: polyethyleneimine (PEI) linear MW 25 kDa (Polysciences), Retro-concentin (System Biosciences), Raltegravir (Selleckchem), Polybrene, Histopaque-1077 (Sigma-Aldrich), Trizol reagent, the SuperScript IV First-Strand Synthesis System, TaqMan Gene Expression Master Mix Dynabeads Untouched Human CD4 T Cells Kit (Thermo Fisher Scientific).

#### HIV-1 mRNA quantitation.

Relative HIV-1 mRNA amounts were determined by modifying the method described by Bullen *et al* [20]. Total RNA from 3~5 × 10^5^ resting T cells or 2.5~5 × 10^6^ patient cells were isolated using Trizol reagent and cDNAs were produced from the RNAs with random hexamer using the SuperScript IV First-Strand Synthesis System (Thermo Fisher Scientific). qPCR was performed with TaqMan Gene Expression Master Mix using HIV-1 3’LTR-specific primers CAGATGCTGCATATAAGCAGCTG and TTTTTTTTTTTTTTTTTTTTTTTTGAAGCAC and probe FAM-CCTGTACTGGGTCTCTCTGG-Iowa black [20]. Duplicate reactions were set up for each sample. After 50~60 cycles of amplification, each 2^-Ct^ average from the duplicate was normalized by the cell number. Obtained values were then normalized to values obtained from the mock treated cells.

### Cell treatments

The following drugs were used in cell treatment experiments: ionomycin, phorbol 12-myristate 13-acetate (PMA), Prostratin, Ingenol-3-angelate (I3A), N,N′-Hexamethylene bis(acetamide) (HMBA), TNFα (Sigma-Aldrich), anti-CD3/anti-CD28 coated magnetic beads (Thermo Fisher), recombinant human interleukin-2 (IL-2, PeproTech), Chidamide, pyroxamide (+)-JQ1 (Cayman Chemical), Bryostatin-1 (Calbiochem), Velcade (Selleck Chemical),GS143 (Tocris), Inhibitors BAY117085, LY2409881, and SC-514 (Selleckchem), Ethacrynic acid (Sigma). HIV-1 integrase inhibitor Raltegravir (Selleckchem) was used as co-treatment in all cell stimulation experiments using HIV-1 latency models.

### Primary cell isolation

PBMCs were isolated from unidentified healthy donor leukocytes (New York Blood Center) using Ficoll separation: 10 mL of blood was mixed with 20 mL 1x PBS pH 7.4, after which 15mL Histopaque-1077 (Sigma-Aldrich) a solution of polysucrose and sodium diatrizoate with a density of 1.077 g/mL, was added to the bottom of the tube. It was then centrifuged at 800 *g* for 20 minutes with the brake set at 5 using a Sorvall Legend X1R, after which the buffy coats were collected, the volume brought up to 45 mL with 1x PBS pH 7.4, and spun at 300 *g* for 10 minutes, and then washed again with PBS and spun. Residual red blood cells were removed by resuspension in ACK Lysing Buffer (Thermo Fisher), an ammonium-chloride-potassium solution, and incubated for 2 minutes at room temperature, then washed twice with 1x PBS pH 7.4.

Dynabeads Untouched Human CD4 T Cells kit (Thermo Fisher) was used to isolate primary resting CD4+ T cells via negative selection. To remove activated CD4 T cells, anti-CD25 antibody was added to the antibody cocktail provided by the kit.: 2x10^8^ PBMCs were resuspended in 2 mL isolation buffer (1x PBS pH 7.4, 2% FBS, 2 mM EDTA), 400 µL FBS, 400 µL mouse IgG antibody mix recognizing CD8, CD14, CD16a and CD16b, CD19, CD36, CD56, CDw123, and CD235a, and 5 µL mouse IgG anti-CD25 (Thermo Fisher), mixed, and incubated for 20 minutes at 4°C. 10 mL isolation buffer was added to the cells, followed by centrifugation at 350 *g* for 8 minutes at 4°C. Supernatant was discarded and the cells resuspended in 2 mL isolation buffer. 2 mL of 20 mg/mL Depletion MyOne Dynabeads (super-paramagnetic polymer beads 1 µm in diameter coated with monoclonal human IgG4 anti-mouse IgG antibody recognizing all mouse IgG subclasses and Fc-specific) were mixed with 2 mL isolation buffer and placed on a magnet for 1 minute, the supernatant discarded, removed from the magnet, and resuspended in 2 mL isolation buffer. The beads were then mixed with the cells and incubated for 15 minutes at room temperature with gentle tilting. 10 mL isolation buffer was added to the cells and thoroughly mixed by pipetting ~10 times using a pipette with a narrow tip opening, avoiding foaming. The tube was placed on a magnet for 2 minutes and the supernatant transferred to a new tube. Then 10 mL isolation buffer was added to the tube containing the beads, and mixed as above by pipetting, then placed on the magnet for 2 minutes and supernatant removed to a new tube. Both supernatant-containing tubes were placed on the magnet one final time for 2 minutes, to remove any residual beads, then the supernatants were collected and pooled together.

### Cell surface staining

2x10^5^ resting CD4+ T cells were resuspended in 100 µL media in 96-well plates, treated at 37°C for 48 hours with: aCD3/aCD28 beads and 100 U/mL IL-2, 5-20 µM GS143, 0.5 µM Prostratin, 5 nM Bryostatin-1, or 5 nM Ingenol-3-angelate, GS143 10 µM alone and in combination with either JQ1 5 µM or Chidamide 5 µM, then collected and washed with 2 mL staining buffer (1x PBS pH 7.4, 2% FBS, 2 mM EDTA), resuspended in 100 µL buffer and incubated for 30 min at 4°C with either of the following FITC-conjugated antibodies: HLA-DR (Class II), CD69 (Thermo Fisher), or CD25 (BD Biosciences). They were then washed with 1 mL staining buffer and centrifuged at 600x *g* for 10 minutes, three times, before being resuspended in 1x PBS pH 7.4 and fixed with 1% formaldehyde for 5 minutes, then washed with 1 mL 1x PBS pH 7.4 and resuspended in 100 µL 1x PBS pH 7.4. They were then analyzed using an Accuri C6 Flow Cytometer (BD Biosciences) to look at the percentage of cells positive for FITC signal.

### Toxicity

5x10^4^ resting CD4+ T cells were resuspended in 100 µL media in 96-well plates and treated with the same compounds as above, along with: 2.5-5 µM Chidamide and 0.5-5µ M JQ1 both alone and in combination with 10 µM GS143, for 48 hours at 37°C. Cell viability was then assessed using the CellTiter-Glo Luminescent Cell Viability Assay (Promega). In this assay, the plate was incubated at room temperature for 30 minutes, 100 µL assay solution was then added to every well, and the plate was placed in a GloMax Discover (Promega) where it was shaken for 2 minutes, incubated at room temperature for 10 minutes, and the luminescent signal detected with an integration time of 1 second.

### Primary cell model

Virus was produced using polyethyleneimine (PEI) (Polysciences) transfection. More specifically, six 100 mM tissue culture dishes of HEK293T cells at 90% confluence were divided 1:2. The next day, transfection mix was prepared for each dish using 1 mL Opti-MEM Reduced Serum Media (Thermo Fisher Scientific), 12 µg of gGn-p6* virus plasmid, 3 µg pcCNA-VPXsiv, and 45 µg PEI. This mix was incubated for 15 minutes at room temperature and then added to the cells. The medium was harvested 48 hours later, filtered through a 0.45 µm filter, and mixed with Retroconcentin (System Biosciences) and incubated at 4°C. After 48 hours, the viral mixture was spun at 1500 g for 30 minutes at 4°C; the viral pellet was resuspended in RPMI-1640 and frozen in liquid nitrogen.

4-5x10^6^ resting CD4+ T cells were resuspended in 500 μL concentrated gGn-p6* virus with 16 µg/mL polybrene, in 24-well plates, and spun at 1,200 g for 1.5 hours at room temperature, then put in a 37°C incubator for 2 hours. Afterwards, RPMI-1640 medium with FBS was added, and the cells then transferred onto the H80 feeder cell line, and incubated overnight. For some experiments, transwell-inserts with a pore size of 1 µm (VWR) were used to allow for separated co-culturing with H80 cells. After 72 hours of co-culture, the infected resting cells were then removed, resuspended in 96-well plates, and incubated with select compounds in the presence of Raltegravir (10 µM) to prevent re-infection for 48 hours, while remaining in co-culture followed by flow cytometry. For RNA analysis, latent cells were cultured in 24-well plates without feeder layer and incubated with different compounds in the presence of Raltegravir for 48 h followed by RNA analysis.

### GFP reading

After 48 hours of treatment, the infected resting CD4+ T cells were fixed in 1% formaldehyde for 5 minutes at room temperature, washed and resuspended in 100 µL 1x PBS pH 7.4 and then mean channel fluorescence (MCF) of GFP was measured. GFP fluorescence levels were measured in 10,000 cells using an Accuri C6 Flow Cytometer (BD Biosciences, Inc., San Jose, CA, USA). A two-parameter gating strategy, which gates live cells according to forward scatter (FSC) and side scatter (SSC), was used to distinguish GFP-derived fluorescence from background. Gating by size and granularity was used to exclude any detached H80 cells. A gate (P2) containing GFP-positive cells was drawn and compared with GFP-negative cells (P1) in terms of cell percentage and MCF using BD Accuri C6 software. Flow cytometry scatter plots showing the purity of resting cells is shown in S3 Fig A and B. Representative plots showing the expression of GFP before (negative control nc) and after stimulation with 10 µM GS143 in infected resting cell are shown in S3 Fig (C-F). Some GFP positive cells were visible in infected samples before treatment with GS143 (S3, D), but not in uninfected cells (S3, B). Notably, both the number and the MCF of GFP-positive cells increased after treatment with GS143 (S3 Fig F).

### Blood specimens from individuals living with HIV-1

Eligible subjects with HIV-1 age 21 to 65 on highly active antiretroviral therapy (HAART), with CD4+ T cell counts greater than 350 per µl and viral loads below 50 copies per ml for at least 5 years and many for 10 years or more were recruited and, following informed consent, enrolled at IDCare in Hillsborough, NJ, through a protocol approved by the Rutgers University Institutional Review Board for ethical approval and Helsinki compliance. Buffy coats were separated from 180 ml whole blood by 1,200 g centrifugation and resting CD4+ T cells were isolated using the same procedure used for healthy donor blood. 2.5-5 x 10^6^ resting CD4+ T cells in 500 µl medium were treated with each LRA for 24 hours and total RNA was then isolated.

### Western blotting

For whole cell lysates, resting CD4+ T cells (1-2 x 10^6^) were placed in 24-well plates and treated with GS143 for different time points. To prepare whole cell lysates, cells were lysed by incubating for 30 min on ice in RIPA (radio immunoprecipitation assay) lysis buffer system (Santa Cruz Biotechnology, Dallas, TX) containing 2 mM PMSF, protease inhibitor cocktail, and 1 mM sodium orthovanadate and supernatants were collected following centrifugation at 10,000 g for 10 min at 4ºC.

For the preparation of cytoplasmic and nuclear lysates, resting CD4+ T cells (1-3 x 10^7^) were plated out onto 6 well culture plates and treated with GS143. Following incubation for desired time, cells were washed with cold PBS. Cells were incubated in ice cold Buffer A (10 mM HEPES, pH 7.9, 10 mM KCl, 1 mM EDTA, 1 mM Dithiothreitol, 1 mM PMSF and Protease Inhibitor Cocktail (Sigma Aldrich, P-2714) by pipeting up and down several times and incubated on ice for 15 min. Next, 10% Nonidet P-40 was added to a final concentration of 0.5% and vortexed for 10 s. Cytoplasmic extracts were collected after pelleting nuclei by centrifugation at 3,000 rpm (approx. 1,000 x g) at 4 ºC for 15 min. Nuclei were washed with Buffer A twice and resuspended in Buffer B (20 mM HEPES, pH 7.9, 400 mM KCl, 1 mM EDTA, 1 mM dithiothreitol, 1 mM PMSF and Protease inhibitors and incubated for 30 min on ice. After centrifugation at 14,000 x g for 15 min at 4 ºC, supernatants were collected as nuclear extracts. Protein concentrations of all extracts were determined by BioRad protein assay. Sample buffer (BioRad Cat#161-0747) was added to the lysates and boiled 5 min and stored at -20ºC. Equivalent amount of protein lysates was analyzed on 10% SDS/PAGE in Tris-Glycine buffer and transferred onto PVDF membrane. After blocking in 5% milk in TBST (150 mM NaCl, 50 mM Tris-HCl, pH 7.5, 0.05% Tween 20) for 1 hour at room temperature, membranes were incubated with appropriate antibody overnight at 4ºC. Following incubation, membranes were washed 3-4 times in TBST and incubated with HRP-conjugated secondary antibody at room temperature for 1 hour. After washing four times in TBST, protein specific bands were visualized with enhanced chemiluminescence substrate *Western Lightning Plus*-*ECL* (Thermofisher). Antibodies against IκB-α, β-catenin, P-β-catenin, P-IKKα/β, p65, cIAP2, XIAP, Bcl2, NIK, LEF-1 and GAPDH were purchased from Cell Signaling Technologies, Danver, MA).

### Expansion of T cells and Immunoprecipitation

PBMC were cultured for 3 days with 5 µg/ml PHA. Cells were collected by scraping and centrifugation and washed twice with PBS. Cells were cultured in RPMI + 10% FBS + P/S supplemented with 300 U/ml of human recombinant (IL2, Peprotech) for 9 days. Cells were maintained 18-24 h without IL-2 [46]. Cytoplasmic and nuclear extracts were prepared as described above. For immunoprecipitation, p65 antibody (1:100 dilution) was added to 200 µl (0.5 -1 mg/ml) lysate in a 1.5 ml Eppendorf tube and incubated overnight at 4ºC with rocking. 20 µl Protein A/G PLUS-Agarose (sc-2003) was added and incubated 2 h at 4ºC on the rocker platform. Following incubation, beads were collected by centrifugation at 2,500 rpm for 5 min at 4ºC. After extensive washing with RIPA lysis buffer (no inhibitor), immunoprecipitates were resuspended in 100 µl sample buffer and boiled for 3 min at 95ºC. Immune complexes were analyzed by SDS-PAGE and western blotting.

### Real-time quantitative PCR to detect NFκB and β-catenin targets

Total RNA was isolated using RNeasy kit (Qiagen). Following the manufacturer protocol. RNA was transcribed to cDNA using High-Capacity cDNA Reverse Transcription Kit (Thermofisher). cDNA was diluted and used for qPCR using SYBR green as a reporter (Power SYBR Green PCR Master Mix, Thermofisher). Reactions were performed in triplicate and quantified by ΔΔC_t_ method. C_t_ values of target genes were normalized to 18S rRNA. Relative mRNA expression was calculated by comparing ΔC_t_ of target to the control.

Primers sets are as follows: *Cyc D1*: forward 5′ GCGAGGAACAGAAGTGC 3′, reverse 5′ GAGTTGTCGGTGTAGATGC 3′; *cMyc*: forward: 5′ CCTGGTGCTCCATGAGGAGAC 3′, reverse 5′ AGACTCTGACCTTTTGCCAGG 3′; *IL6*: forward 5′ AGACAGCCACTCACCTCTTCA 3′, reverse 5′ CACCAGGCAAGTCTCCTCATT 3′; *TNFA*: forward 5′ GTGCTTGTTCCTCAGCCTCT T 3′, reverse 5′ ATGGGCTACAGGCTTGTCATC 3′; *IL1b*: forward 5′ GAAGCTGATGGCCCTAAACAG 3′, reverse 5′ AGCATCTTCCTCAGCTTGTCC 3′; *IL8*: forward 5′ ACTGAGAGTGATTGAGAGTGGAC 3′, reverse 5′ AACCCTCTGCACCCAGTTTTC 3′

IkBA:forward 5′- ATGTTCCAGGCGGCCGAG-3′, reverse 5′- TGCAGGAACGAGTCCCCG-3′, 18s: forward 5′CGGCTACCACATCCAAGGAA3′, Reverse 5′ GCTGGAATTACCGCGGCT3′; ICAM: forward 5′AGCGGCTGACGTGTGCAGTAAT3′, reverse TCTGAGACCTCTGGCTTCGTCA3′; AXIN2: forward 5′GAGTGGACTTGTGCCGACTTCA3′, reverse 5′GGTGGCTGGTGCAAAGACATAG3′ and MMP9: forward-5′GCCACTACTGTGCCTTTGAGTC3′, reverse 5′ CCCTCAGAGAATCGCCAGTACT3′

#### Bliss independence analysis.

Bliss independence analysis [64] was employed to determine the synergy of GS143 with other LRA in latency reversal resting CD4+ T cells infected with gGn-p6* were treated with indicated compounds for 48 h. Base line fluorescence from untreated cells (NC) was subtracted from mean channel fluorescence (MCF) of treated cells and normalized with that of the positive control to determine the fraction affected fa_x_ = (MCF_drug x_ -MCF_NC_)/ (MCF_positive control_ – MCF_NC_). Since some of our combinations gave a stronger signal than the positive control PMA + Ionomycin, we used highest MCF + 1 in each experiment to serve as a positive control. For drugs x and y, the predicted fraction fa_xy_P is calculated as the equation fa_xy_P= fa_x_ + fa_y_ –(fa_x_)(fa_y_). The observed combination value (fa_xy_O, calculated as MCF_xy_ -MCF_NC_)/ (MCF_positive control_ – MCF_NC_) indicates the observed fraction affected with a combination of drugs x and y. The equation Δfa_xy_ = fa_xy_O - fa_xy_P was used to define synergy if Δfa_xy_ > 0, additive if Δfa_xy_ = 0 or antagonism if Δfa_xy_ < 0. Statistical significance was determined using a two tailed t-test between fa_xy_O and fa_xy_P, where p ≤0.05 was considered significant. Synergy was calculated using the data shown in Fig 4A.

#### Statistical analysis.

GraphPad Prism 9.4 was used for statistical analysis. One-way ANOVA was performed to compare the overall effects of different LRAs vs. untreated control. Dunnett’s multiple comparisons were then used to determine statistical difference between each LRA and untreated control. HIV-1 LTR mRNA fold changes were presented as medians, interquartile ranges (IQR) and minimum and maximum, and Wilcoxon matched-pairs signed rank test (two tailed) was used to determine the non-parametric differences. Target genes’ mRNA fold changes (mean ± SEM) were analyzed by two-tailed t-test assuming equal variance. *P* value less than 0.05 was considered statistically significant.

## Supporting information

S1 Data All data used for statistical analyses and generation of graphs in Figs 1A, 1C, 2, 3, 4A, 5B and 8. (XLSX)

S1 Fig Protocol for establishing latent virus infection in primary resting CD4+ T cells. (A) Primary resting CD4+ T cells were isolated from leukocyte-enriched healthy donor samples using a Ficoll gradient and negative selection. The isolated T cells were infected by spinoculation with gGn-p6* virus containing Vpx protein. Three days post infection cultures were treated with test compounds followed 48 h later by flow cytometric analyses. Where indicated, resting cells were co-cultured with the H80 glioma cell line at day-1 post infection. (B) Structural formula of GS143.
(TIF)

S2 Fig Immunoblotting showing LEF/TCF and CD4 expression. (A) Expression of LEF/TCF family of proteins in primary resting CD4+ T cells. Whole cell extracts from primary resting CD4+ T cells, HeLa and 293T cells were analyzed by immunoblotting with TCF1, TCF3, TCF4, LEF1 and GAPDH antibodies. (B) Expression of CD4 in primary resting CD4+ T cells. Whole cell extracts from primary resting CD4+ T cells treated with GS143 and analyzed by immunoblotting with CD4 and GAPDH antibodies.
(TIF)

S3 Fig Flow cytometry scatter plots. A and B show the purity of resting cells. Representative plots showing the expression of GFP in infected resting CD4+ T cells before (C, D; negative control nc) and after stimulation with 10 µM GS143 (E, F).
(TIF)

S4 Fig Supporting information for Fig 5. IκBɑ and β-catenin specific bands were quantitated by ImageJ and the percentage of normalized IκBɑ (A and B) and β-catenin (C and D) for each condition were plotted as a function of GS143 concentration from 3-6 independent experiments (each subject is color coded within each panel) involving different subjects. Data are presented as medians, interquartile ranges (IQR) and minimum and maximum. Statistical significances (*p*≤ 0.05) of GS143 treated relative to untreated control resting CD4+ T cells were analyzed by t test (1-tailed pairwise comparison) and *p*-values and the number of independent observations per time point are shown.
(TIF)

S5 Fig Supporting information for Fig 6. cIAP2, NIK, P-IKKɑ/β, P-S536-p65 and XIAP specific bands were quantitated by ImageJ and the percentage of normalized protein level for each condition were plotted as a function of GS143 concentration from 2-5 independent experiments involving different subjects (each subject is color coded within each panel and not the same subjects used for all panels). Data are presented as medians, interquartile ranges (IQR) and minimum and maximum. Statistical significances (*p*≤ 0.05) of GS143 treated relative to the level in untreated control resting CD4+ cells were analyzed by t test (1-tailed pairwise comparison) and *p*-values and the number of independent observations per time point are shown. Panels A-D and F are related to Fig 6 (immunoblotting with whole cell lysates) and panel E is related to Fig 7B (immunoblotting with cytoplasmic lysates).
(TIF)
