# Supplementary figures and images for: GS143, an inhibitor of E3 ligase β-TrCP, reverses HIV-1 latency without activating T cells via unconventional activation of NFκB
Source: PLoS Pathog. 2025 Apr 1;21(4):e1013018. doi: 10.1371/journal.ppat.1013018 (PMC11999137; doi:10.1371/journal.ppat.1013018)

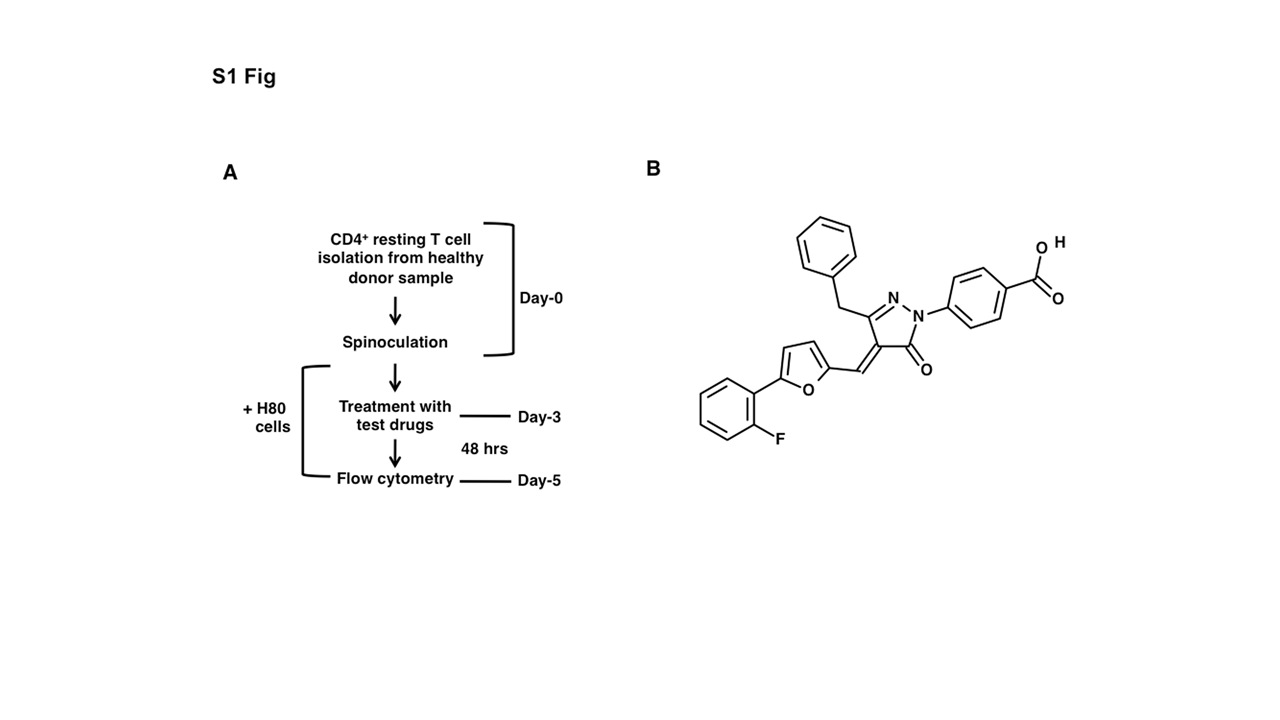

Supplement: S1 Fig — (A) Primary resting CD4+ T cells were isolated from leukocyte-enriched healthy donor samples using a Ficoll gradient and negative selection. The isolated T cells were infected by spinoculation with gGn-p6* virus containing Vpx protein. Three days post infection cultures were treated with test compounds followed 48 h later by flow cytometric analyses. Where indicated, resting cells were co-cultured with the H80 glioma cell line at day-1 post infection. (B) Structural formula of GS143. (TIF) [file ppat.1013018.s002.TIF]

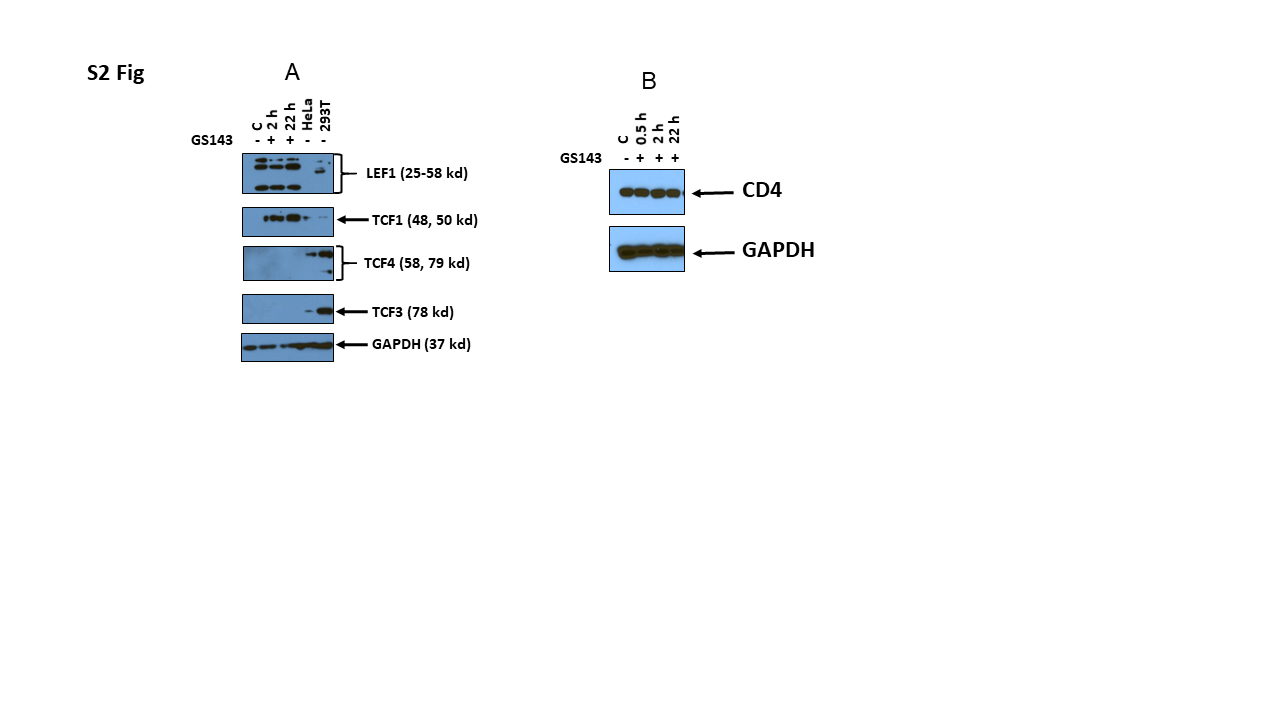

Supplement: S2 Fig — (A) Expression of LEF/TCF family of proteins in primary resting CD4+ T cells. Whole cell extracts from primary resting CD4+ T cells, HeLa and 293T cells were analyzed by immunoblotting with TCF1, TCF3, TCF4, LEF1 and GAPDH antibodies. (B) Expression of CD4 in primary resting CD4+ T cells. Whole cell extracts from primary resting CD4+ T cells treated with GS143 and analyzed by immunoblotting with CD4 and GAPDH antibodies. (TIF) [file ppat.1013018.s003.TIF]

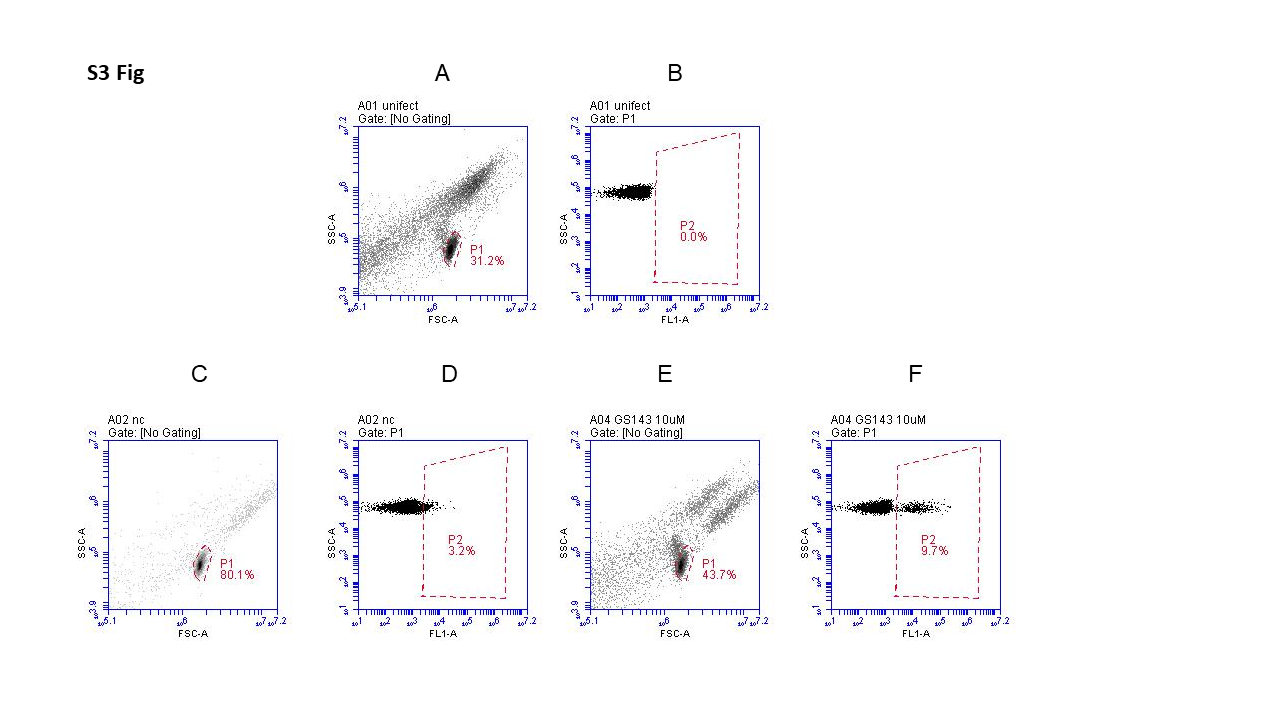

Supplement: S3 Fig — A and B show the purity of resting cells. Representative plots showing the expression of GFP in infected resting CD4+ T cells before (C, D; negative control nc) and after stimulation with 10 µM GS143 (E, F). (TIF) [file ppat.1013018.s004.TIF]

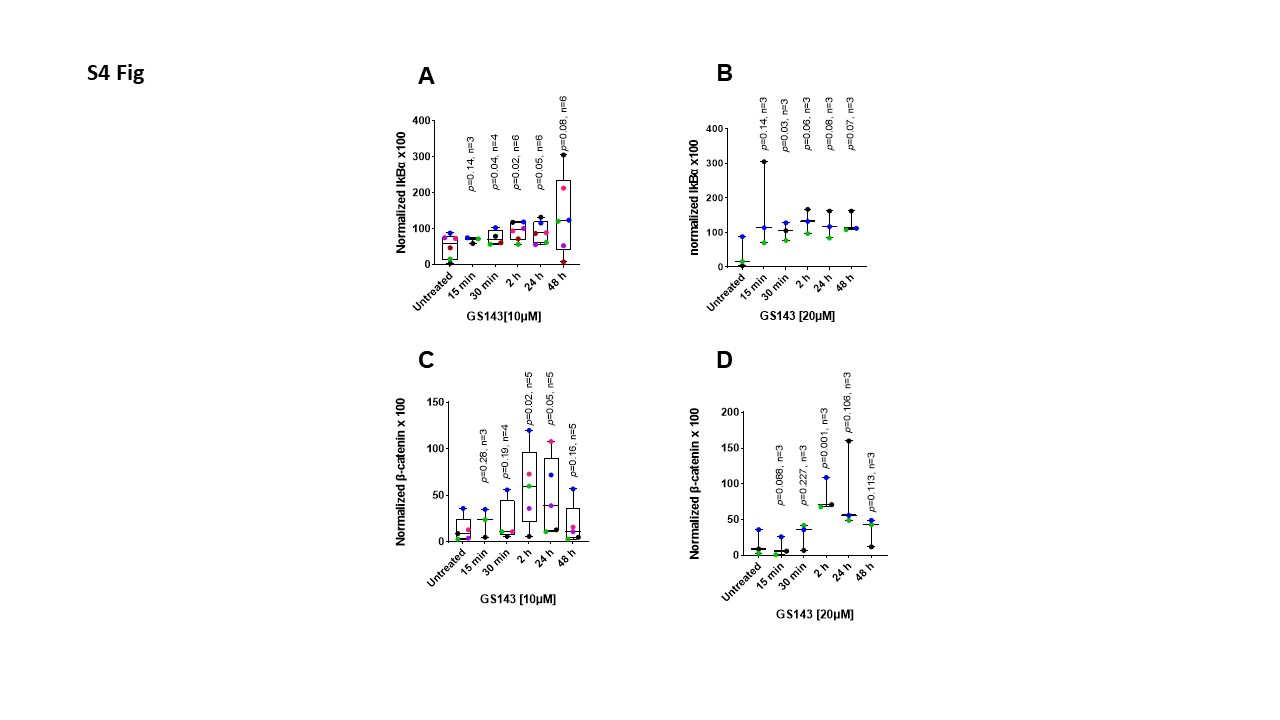

Supplement: S4 Fig — IκBɑ and β-catenin specific bands were quantitated by ImageJ and the percentage of normalized IκBɑ (A and B) and β-catenin (C and D) for each condition were plotted as a function of GS143 concentration from 3-6 independent experiments (each subject is color coded within each panel) involving different subjects. Data are presented as medians, interquartile ranges (IQR) and minimum and maximum. Statistical significances (p≤ 0.05) of GS143 treated relative to untreated control resting CD4+ T cells were analyzed by t test (1-tailed pairwise comparison) and p-values and the number of independent observations per time point are shown. (TIF) [file ppat.1013018.s005.TIF]

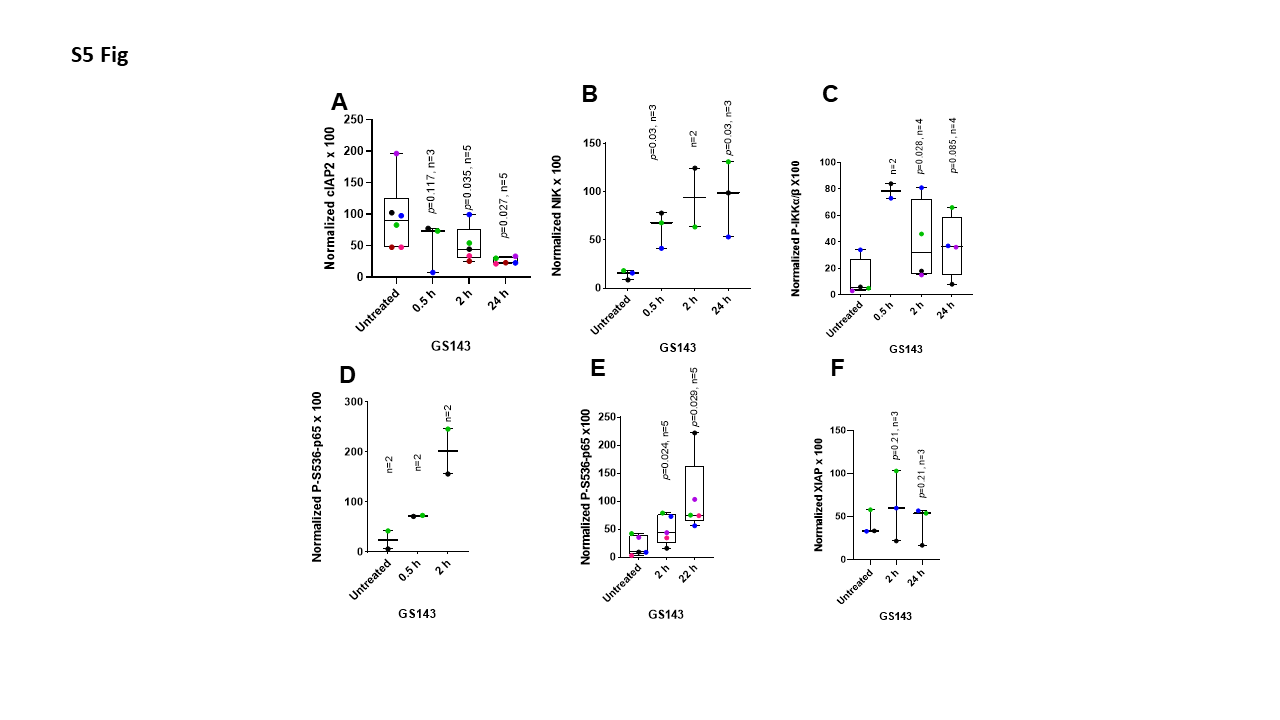

Supplement: S5 Fig — cIAP2, NIK, P-IKKɑ/β, P-S536-p65 and XIAP specific bands were quantitated by ImageJ and the percentage of normalized protein level for each condition were plotted as a function of GS143 concentration from 2-5 independent experiments involving different subjects (each subject is color coded within each panel and not the same subjects used for all panels). Data are presented as medians, interquartile ranges (IQR) and minimum and maximum. Statistical significances (p≤ 0.05) of GS143 treated relative to the level in untreated control resting CD4+ cells were analyzed by t test (1-tailed pairwise comparison) and p-values and the number of independent observations per time point are shown. Panels A-D and F are related to Fig 6 (immunoblotting with whole cell lysates) and panel E is related to Fig 7B (immunoblotting with cytoplasmic lysates). (TIF) [file ppat.1013018.s006.TIF]
